# Fractionation of the visuomotor feedback response to directions of movement and perturbation

**DOI:** 10.1152/jn.00377.2013

**Published:** 2014-08-06

**Authors:** David W. Franklin, Sae Franklin, Daniel M. Wolpert

**Affiliations:** Computational and Biological Learning Lab, Department of Engineering, University of Cambridge, Cambridge, United Kingdom

**Keywords:** adaptive control of reflex magnitude, motor control, online control, reflex modulation, visually guided reaching

## Abstract

Recent studies have highlighted the modulation and control of feedback gains as support for optimal feedback control. While many experiments contrast feedback gains across different environments, only a few have demonstrated the appropriate modulation of feedback gains from one movement to the next. Here we extend previous work by examining whether different visuomotor feedback gains can be learned for different directions of movement or perturbation directions in the same posture. To do this we measure visuomotor responses (involuntary motor responses to shifts in the visual feedback of the hand) during reaching movements. Previous work has demonstrated that these feedback responses can be modulated depending on the statistical distributions of the environment. Specifically, feedback gains were upregulated for task-relevant environments and downregulated for task-irrelevant environments. Using these two statistical distributions, the first experiment examined whether these feedback responses could be independently modulated for the same limb posture for two directions of movement (same limb posture but on either an inward or outward movement), while the second examined whether the feedback responses could modulate, within a single movement, to perturbations to the left or right of the reach. Both experiments demonstrated that visuomotor feedback responses could be learned independently such that the response was appropriate for the environment. This work demonstrates that feedback gains can be simultaneously tuned (upregulated and downregulated) depending on the state of the body and the environment. The results indicate the degree to which feedback responses can be fractionated in order to adapt to the world.

recent work has highlighted the ability of the sensorimotor control system to modulate the feedback responses according to the environment. For example, feedback gains have been shown to modulate according to the environmental dynamics (Cluff and Scott 2013; Franklin et al. 2007, 2012; Kimura and Gomi 2009), the limb dynamics (Kurtzer et al. 2008; Pruszynski et al. 2011a), the visual statistics (Franklin and Wolpert 2008), the shape of the target (Knill et al. 2011; Nashed et al. 2012), and when the two limbs manipulate a single object (Diedrichsen 2007; Dimitriou et al. 2012; Omrani et al. 2013). Such modulation has been used to support the theory that the skillful movement occurs through the setting of the appropriate feedback gains to the task being performed, a theory termed optimal feedback control (Liu and Todorov 2007; Scott 2004; Todorov 2004; Todorov and Jordan 2002). Although most studies have examined differences in the feedback responses in a blocked design (e.g., Ahmadi-Pajouh et al. 2012; Nashed et al. 2012), several papers have recently demonstrated modulation of these responses within a single trial (Dimitriou et al. 2013; Kimura and Gomi 2009; Knill et al. 2011). Dimitriou and colleagues (2013) demonstrated an online update in the feedback gain due to changes in the location of the reach target. By using targets with different shapes, Knill et al. (2011) showed that the visuomotor feedback gains can be modulated differently for perturbations in the direction of the reach compared with perturbations laterally. Kimura and Gomi (2009) measured the stretch response to mechanical perturbations, showing that magnitude was modulated early in a movement depending on the force field that would be applied later in the movement. This work showed that subjects were able to select the appropriate stretch feedback gain, different for perturbations to one side of the movement or the other, based on the task being performed and to adapt this from one trial to the next. Here we examined whether such differential modulation of feedback gains for perturbation direction is also possible for visuomotor perturbations. Although the work of Knill and colleagues (2011) shows modulation of feedback gains between direction and extent for different target shapes, as the targets were symmetrical about the two principal axes it was not possible to examine whether gains could be modulated within a single axis for two directions of perturbation. Here we investigate whether visuomotor feedback gains can also be modulated independently to the left or right of a reaching movement for the same limb posture (i.e., for identical limb posture and distance to the target). Such a differential response would suggest either that an optimal controller sets independent feedback gains to errors on one side of the movement or the other or that the controller switches between multiple optimal controllers.

Rapid motor responses to visual signals occur in response to the presentation of a visual stimulus (Corneil et al. 2004, 2007) and shifts in the target location (Prablanc and Martin 1992), the entire background (Saijo et al. 2005), and the representation of the hand position (Brenner and Smeets 2003; Sarlegna et al. 2003; Saunders and Knill 2003). The visually induced corrective responses occur relatively quickly (150 ms) after the representation of the hand or target moves and do not require subjects to consciously perceive these movements (Goodale et al. 1986; Prablanc and Martin 1992). Early components of all of these visually induced motor responses have been shown to be involuntary in nature (Day and Lyon 2000; Franklin and Wolpert 2008; Gomi et al. 2006; Saijo et al. 2005).

Previously we showed that this visuomotor reflex can be tuned to the statistical properties of the environment (Franklin and Wolpert 2008). In particular, we found an increase in the magnitude of the response when the environment contained task-relevant sensory discrepancies and a reduction in gain when the environment contained task-irrelevant sensory discrepancies. The sensory discrepancy environments were produced by smoothly moving the visual feedback away from the actual hand position in the middle of the movement. In the task-relevant condition, these discrepancies were maintained and therefore required correction, while in the task-irrelevant condition these discrepancies were smoothly removed such that the visual feedback was matched with the actual hand position at the end of the movement. In the present study we exploit these findings to examine whether the sensorimotor control system can control the magnitude or gain of the reflex response simultaneously to both task-irrelevant and task-relevant sensory discrepancies. First we determine whether the visuomotor reflex can be tuned differently in the same spatial location but with movements of different directions. Specifically, task-relevant discrepancies are applied for one direction of movement and task-irrelevant discrepancies for the other. Second, we examine whether the feedback responses can be tuned independently to perturbations on the right and on the left of the reaching movement by varying the task relevancy of sensory discrepancies on either side of the reaching movement (with task-irrelevant discrepancies on one side and task-relevant discrepancies on the other). We demonstrate that the sensorimotor control system can tune the reflex gain simultaneously in a particular limb posture to different states of the limb prior to the perturbation and to different perturbation directions.

## MATERIALS AND METHODS

Twenty-four subjects participated in 2 groups of 12 subjects for the first experiment: *group 1* (4 women and 8 men; mean ± SD age: 23.9 ± 5.8 yr) and *group 2* (7 women and 5 men; age: 23.8 ± 4.8 yr). Sixteen subjects (10 men and 6 women; mean age: 25.1 ± 6.5 yr), six of whom also participated in the first experiment, were recruited to participate in the second experiment. All subjects were right-handed according to the Edinburgh handedness inventory (Oldfield 1971), with no reported neurological disorders and normal or corrected to normal vision. Subjects gave informed consent, and the experiments were approved by the institutional ethics committee.

### 

#### Experimental setup.

**Fig. 1.**
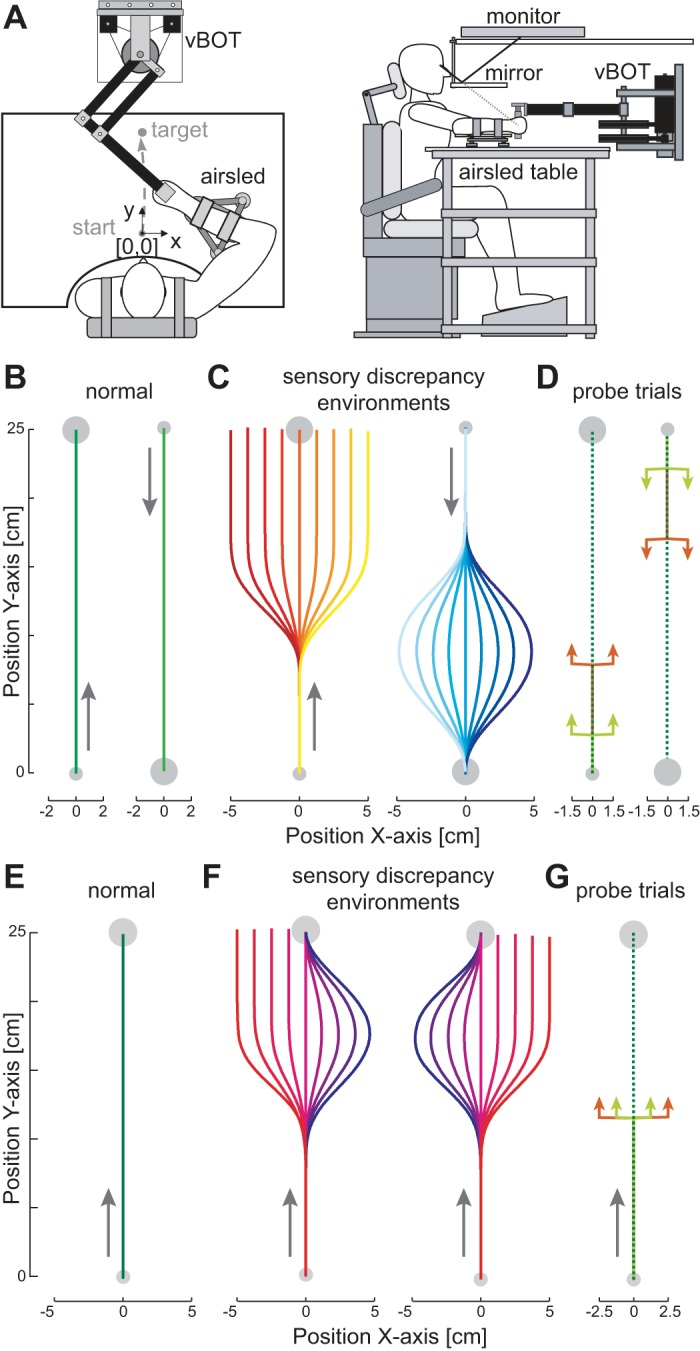
The experimental setup. *A*: the subject grasps the robotic manipulandum (vBOT) while seated. Visual feedback is presented veridically with a top-mounted computer screen viewed through a mirror. The subject's forearm is fixed to and supported by an airsled. *B*: *experiment 1*: effect of movement direction. Subjects made movements alternately in 2 directions (outward and inward). Initially, movements were performed in the normal condition, where the cursor reproduced the hand trajectory exactly. Note that for clarity of display the outward and inward movements have been horizontally offset in the plots. *C*: in the second phase of the experiment, the outward movement was performed in the task-relevant environment (red-yellow) whereas the return movements were performed in the task-irrelevant environment (cyan-blue). A second group of subjects performed experiments in which these directions were reversed. In the task-relevant condition, the visual cursor smoothly moved away from the hand trajectory to 1 of 9 amplitudes (including 0) and remained at this point for the rest of the movement. In the task-irrelevant condition, the visual cursor smoothly moved away from the hand trajectory exactly as in the task-relevant condition but then returned smoothly such that it agreed with the physical hand position at the end of the movement. *D*: the visual perturbations (probe trials) used in *experiment 1* to examine the magnitude of the visually induced motor response. On random trials, the hand was mechanically constrained to a straight-line trajectory to the target and the visual cursor representing the hand was jumped laterally away from the actual hand position and returned 250 ms later. Two different onsets of the perturbations (light green and orange arrows) were chosen for each direction: 1 pair (orange) were matched for the spatial location of the visual perturbation of the 2 directions of movement (matched stimuli perturbations: occurring at 30% of the distance to the target), and the other pair (light green) were matched so that the visuomotor response occurred at a similar location for the 2 directions of movement (matched response perturbations: occurring at 10% of the distance to the target). *E*: *experiment 2*: effect of perturbation direction. Subjects performed outward reaching movements. Initially, all subjects performed movements in the normal environment. *F*: in the second half of the experiment 1 group of subject made movements under 1 of the sensory discrepancy environments (*left*), whereas the other group was presented with the opposite environment (*right*). In these environments, the visual cursor representing the subject's hand was manipulated in a task-relevant manner on one side of the movement and in a task-irrelevant manner on the other side of the movement. *G*: the visual perturbations (probe trials) used in *experiment 2* to examine the magnitude of the visually induced motor response. Two sizes of perturbation were used on either side of the zero perturbation to examine changes in the gain of the response.

Movements investigated in this study were right-handed outward (away from the body) and inward (toward the body) movements in the horizontal plane at ∼10 cm below the subjects' shoulder level. The forearm was supported against gravity with an airsled. The handle of the robotic manipulandum (vBOT) used to generate the environmental dynamics (null field or mechanical channels) was grasped by the subject (Fig. 1*A*). Position and force data were sampled at 1 kHz. End-point forces at the handle were measured with an ATI Nano 25 six-axis force-torque transducer (ATI Industrial Automation). Visual feedback was provided with a computer monitor mounted above the vBOT and projected veridically to the subject via a mirror. This virtual reality system covers the manipulandum, arm, and hand of the subject, preventing any visual information of their location. Full details of the vBOT have been published previously (Howard et al. 2009). The exact time that the stimuli were presented visually to the subjects was determined by using the video card refresh rate and confirmed with an optical sensor.

Movements were made from a 1.0-cm-diameter start circle to a 2.0-cm-diameter target circle, both of which were centered in front of the subject. The subjects' arm was hidden from view by the virtual reality visual system on which the start and target circles, as well as a 0.6-cm-diameter cursor used to track instantaneous hand position, were projected. The distance between the centers of the start and target circles was 25.0 cm. Successful movements were defined as those that entered the target without overshooting and with movement durations in the range 700 ± 75 ms and were accompanied with feedback (“good” or “great”). When subjects performed successful movements, a counter increased, and subjects were instructed to increase the number of the counter as much as possible throughout the experiment. When subjects performed unsuccessful movements, they were provided with feedback as to why the movement was not considered successful (“too fast,” “too slow,” or “overshot target”). Trials were self paced; subjects initiated a trial by moving the hand cursor into the start circle and holding it within the target for 450 ms. A beep then indicated that the subject could begin the movement to the target. The duration of the movement was determined from the time that the subject's hand exited the start target until the time that the subject's hand entered the final target.

#### Electromyography.

In the second experiment, surface electromyography (EMG) was recorded from two monoarticular shoulder muscles: pectoralis major and posterior deltoid; two biarticular muscles: biceps brachii and long head of the triceps; and two monoarticular elbow muscles: brachioradialis and lateral head of the triceps. EMG was recorded with the Delsys Bagnoli (DE-2.1 Single Differential Electrodes) EMG system (Boston, MA). The electrode locations were chosen to maximize the signal from a particular muscle while avoiding cross talk from other muscles. The skin was cleansed with alcohol and prepared by rubbing an abrasive gel into the skin. This was removed with a cotton pad, and the gelled electrodes were secured to the skin with double-sided tape. The EMG signals were band-pass filtered online through the EMG system (20–450 Hz) and sampled at 2 kHz. The EMG signals are aligned with the force and position signals with a signal from the serial port, which is recorded on a channel with the EMG. This signal is changed at the onset and offset of any perturbation in order to ensure that the correct timing is achieved over the period of interest.

#### Probe trials to measure reflex gain.

Visually induced motor responses were examined with perturbations similar to those previously described (Dimitriou et al. 2013; Franklin et al. 2012; Franklin and Wolpert 2008). In the middle of the movements to the target, the cursor representing the hand position was laterally jumped away from the current hand position, held at a fixed distance from the actual hand trajectory for 250 ms, and then returned to the actual hand position for the rest of the movement. The entire visual perturbation lasted for 250 ms. During such probe trials, the hand was physically constrained to the straight path between the start and final targets [mechanical channel trial (Milner and Franklin 2005; Scheidt et al. 2000) generated by the vBOT]. The mechanical channel was implemented as a stiffness of 5,000 N/m and damping of 2 N·m^−1^·s^−1^ for any movement lateral to the straight line joining the starting location and the middle of the target. This constrains the physical hand location using the channel such that no change in the arm configuration occurs. The force produced in response to the visual perturbation can be measured against the channel wall with the force sensor. As the visual perturbation returns to the actual hand trajectory, subjects do not need to respond to the visual perturbation in order to produce a successful movement to the target. A discussion of the possible concerns with the use of mechanical channels to measure visuomotor feedback gains can be found in the methods section of Dimitriou et al. (2013). These visual perturbations were applied perpendicular to the direction of the movement (either to the left or the right). For comparison, a zero-perturbation trial was also included, in which the hand was held to a straight-line trajectory to the target but the visual cursor remained at the hand position throughout the trial. The perturbation trials were randomly applied during movements in a blocked fashion such that one of each perturbation type (each perturbation direction or onset time) was applied within each block of trials.

### 

#### Experiment 1—effect of movement direction.

Our previous experiment (Franklin and Wolpert 2008) demonstrated that subjects could adapt the magnitude of the reflex response (either increasing or decreasing the reflex response) to two different distributions of sensory discrepancies (task relevant or task irrelevant, respectively) when they were presented on different days. Here we examine whether subjects can simultaneously control their reflex response to the two different distributions when they are presented in the same location but for different directions of movement (Fig. 1*B*). Subjects made both outward and inward movements directly in front of their body. There were two stages to the experiment. The first half of the experiment (comprised of 1,122 movements) was performed in a normal environment, one in there was no discrepancy between the visual path of the hand and the actual physical path of the hand. For the first group of subjects (*n* = 12), the second half of the experiment had task-relevant discrepancies on the outward movements and task-irrelevant discrepancies on the inward movements. A second group of subjects (*n* = 12) had the reverse: task-irrelevant discrepancies on the outward movements and task-relevant discrepancies on the inward movements. In the task-relevant condition, at a point in the trajectory that was 30% of the distance to the target (7.5 cm from the start), the visual cursor representing the hand position underwent a smooth (minimum jerk) movement laterally to the movement direction to a distance from the set [−5.0, −3.75, −2.5, −1.25, 0, 1.25, 2.5, 3.75, 5.0] cm in the subsequent 8.5 cm of the movement distance, remaining at this position laterally for the rest of the movement (Fig. 1*C*, *left*). Subjects were required to determine the appropriate response in order to bring the hand cursor back in to the target and be credited with a successful trial. In this condition, while the lateral change in the visual location of the hand position produces a visuomotor discrepancy, the visual signal provides reliable information about the amount of compensation that the subjects will need to produce in order to successfully complete the movement. In the task-irrelevant condition, 30% of the distance to the final target (7.5 cm from the start), the visual cursor representing the hand position underwent a smooth (minimum jerk) movement laterally to a distance from the set [−5.0, −3.75, −2.5, −1.25, 0, 1.25, 2.5, 3.75, 5.0] cm in the subsequent 8.5 cm of movement and then returned to the actual hand position in the final 8.5 cm of outward movement in the same manner (Fig. 1*C*, *right*). Thus at the final target the hand position and cursor position were matched. While this condition initially produces a visuomotor discrepancy identical to the task-relevant condition, the size of this discrepancy between visual and haptic signals in the middle of the movement does not provide reliable information about the location of the hand at the end of the trial. After each movement, the robotic manipulandum moved the subjects' limb to the start position, while visual feedback of the location of the hand was not presented.

In all environments five different types of visual perturbation trials or probe trials (Fig. 1*D*) were presented within a single block of 14 trials (5 probe trials and 9 nonprobe trials from the distribution) in each direction in order to assess the feedback gains. This means that a single block of movements (28 movements) was made up of 14 outward movements and 14 inward movements. If the nine nonprobe movements were from a distribution of sensory discrepancies, then one of each discrepancy was included in the block of movements. Four of the five probe trials used in this experiment were two 1.5-cm visual displacements either to the left or the right of the movement occurring at either 10% or 30% of the movement distance and lasting for 250 ms. The fifth probe trial had a zero visual displacement for comparison against the shifted visual perturbations. The perturbations occurring at 10% of the movement distance were implemented to examine the response magnitude when the positions at which the force response occurs during the movement were matched for both the outward and inward reaching movements. On the other hand, the perturbations occurring at 30% of the movement distance were implemented to examine the responses when the stimuli (the visual perturbations) were matched for the limb posture in the inward and outward movements. A single nonprobe movement was always performed first in any new environment, such that a probe trial was never the first movement. While lateral movement in the random probe trials was constrained by the mechanical channel, the subjects were free to move in any manner during all of the other trials. To examine the effects of each of the two changed conditions (task relevant or task irrelevant), the responses were contrasted with the normal condition in the same movement direction in order to get an appropriate baseline for both conditions. Subjects performed 40 blocks of movements in each condition. This resulted in 1,122 movements in the normal condition (561 outward and 561 inward) followed by 1,122 movements in the sensory discrepancy environments (561 outward task relevant and 561 inward task irrelevant or vice versa for the second group of subjects). Subjects were required to take short breaks every 300 movements throughout the experiment. They were also allowed to rest at any point they wished by releasing a safety switch on the handle.

#### Experiment 2—effect of perturbation direction.

The second experiment was designed to examine whether the reflex response could be modulated differently for different directions of visual perturbation. To determine whether the reflex gains could be modulated simultaneously, subjects were exposed to a distribution of sensory discrepancies that were all task relevant on one side and task irrelevant on the other side of the reaching movement (Fig. 1*F*). Subjects performed the experiment on two separate days. All subjects started with the normal environment in the first phase of the experiment (Fig. 1*E*); the second phase was performed differently for two randomly chosen groups. On *day 1*, half of the subjects experienced the task-relevant sensory discrepancies on the right side of the reaching movement (positive perturbations) and the task-irrelevant sensory discrepancies on the left side of the reaching movement (negative perturbations) while the other half experienced the opposite mapping of direction to task relevance (Fig. 1*F*). On *day 2* the mapping of direction to task relevance was reversed for all subjects.

Subjects made outward reaching movements to a target centered directly in front of their body. Each day of the experiment had two components. Initially, subjects made reaching movements in a normal environment—one in which the physical location and the visual location of the hand matched throughout the movement. After 50 blocks of this condition were completed, the sensory discrepancy environment was presented. In this environment, at a point in the trajectory that was 40% of the distance to the target (10 cm from the start), the visual cursor representing the hand position underwent a smooth (minimum jerk) movement laterally to the movement direction to a distance from the set [−5.0, −3.75, −2.5, −1.25, 0, 1.25, 2.5, 3.75, 5.0] cm in the next 7.5 cm of the outward movement distance. On perturbations to one side, the displacement was maintained throughout the rest of the movement, requiring subjects to actively respond to this displacement and move the visual location of the hand back toward the target in order to achieve the task. However, for perturbations to the other side, the visual signal of the hand was returned to the actual hand position over the final 7.5 cm of outward movement in the same manner such that at the final target the hand position and cursor position were matched. Note that in both the sensory discrepancy environment and the normal environment, there are trials in which the visual cursor perfectly matches the hand location during the reach (zero sensory discrepancy trials). These trials can be used to examine whether there are any differences in the direction and curvature of the movement between the two conditions.

During both phases of the experiment, five different visual perturbation trials or probe trials (Fig. 1*G*) were presented within a single block of 14 trials (5 probe trials and 9 unperturbed trials from the distribution) in each direction in order to assess the reflex response. The five probe trials consisted of two perturbations to the left [−1.25 cm, −2.5 cm], two perturbations to the right [1.25 cm, 2.5 cm], and a zero perturbation for comparison. The perturbations were initiated at 45% of the distance to the target (11.25 cm into the movement) and lasted for 250 ms. The other nine unperturbed trials contained in each block were either composed of nine normal trials (normal environment) or composed of one of each of the sensory discrepancy trials such that every block contained equal numbers of both the task-relevant and task-irrelevant trials (sensory discrepancy environment). A single movement was always performed first in any new environment, such that a probe trial was never the first movement. While lateral movement in the random probe trials was constrained by the mechanical channel, the subjects were free to move in any manner during all of the other trials. Subjects performed 50 blocks of movements in each phase. This resulted in 701 movements in the normal condition followed by 701 movements in the sensory discrepancy environment. Subjects were required to take short breaks every 300 movements throughout the experiment. They were also allowed to rest at any point they wished by releasing a safety switch on the handle.

#### Analysis.

Analysis of the experimental data was performed with MATLAB R2012a. EMG data were high-pass filtered with a fifth-order, zero phase-lag Butterworth filter with a 30-Hz cutoff and then rectified. Hand acceleration was obtained by double differentiating the position data and low-pass filtering at 15 Hz with a fifth-order, zero phase-lag Butterworth filter after each differentiation. As performed previously (Dimitriou et al. 2013; Franklin and Wolpert 2008), the responses to visual perturbations in the first five blocks of a condition were not used for analysis in order to avoid the possible influence of initial high-gain trials at the beginning of the experiments. Therefore only the results from the last 45 blocks (*blocks 6–50*) are used in any of the analyses and results. Individual probe trials were aligned on visual perturbation onset and averaged across repetitions. The response to the right visual perturbation on probe trials was subtracted from the response to the left perturbation on probe trials in order to provide a single estimate of the motor response to the visual perturbation for the first experiment. In the second experiment, the responses to the right and left perturbations were examined separately by subtracting them from the zero perturbation. In this manner the response to each side could be examined. To examine the rapid response (i.e., “reflex”), we calculated the average postperturbation EMG (120–180 ms) and force (180–230 ms) (Franklin and Wolpert 2008). ANOVAs were examined in SPSS (v. 21) with the general linear model. If a significant main effect was found, Tukey's honestly significant difference (HSD) post hoc test was used to examine differences. Statistical significance was considered at the *P* < 0.05 level for all statistical tests. Specifically, each data point used for statistical analysis represented the average across the reflex time window of a single perturbation trial for EMG and the difference between pairs of trials for force. All statistical tests on the EMG data are performed on unscaled and unsmoothed subject data. For plotting purposes only (Fig. 7), the EMG was scaled, smoothed, and averaged across subjects (but not across muscles). To do this for a particular muscle we calculate a single scalar for each subject that is used to scale the muscle's EMG traces for all trials for that subject. The scalar was chosen so that the mean (across trials) of the EMG data averaged over the period −50 to 50 ms relative to the onset of the perturbation was equal across subjects (and set to the mean over all the subjects). This puts each subject on an equal scale to influence any response seen in the data. Prior to averaging, the individual muscle traces were initially smoothed with a 10-point (5 ms) moving average. Once averaged across all subjects, the mean and SE were determined for each condition and the mean muscle activity was then smoothed with a 10-point (5 ms) moving average prior to plotting.

For both *experiments 1* and *2*, analysis was performed in order to determine the earliest time at which visuomotor responses were modulated independently for the two environments. Specifically, to examine whether there was independent modulation of the feedback responses for different states and to determine at what time such independent modulation occurs, the difference in the force response in the sensory discrepancy environment was calculated every 1 ms relative to the temporal pattern in the normal environment (mean for each subject). *t*-Tests were then used to test whether the difference in these responses was significant between the task-relevant and task-irrelevant environments at each time point. Significant differences were considered at *P* < 0.05, Bonferroni corrected for multiple comparisons.

## RESULTS

### 

#### Experiment 1—effect of movement direction.

**Fig. 2.**
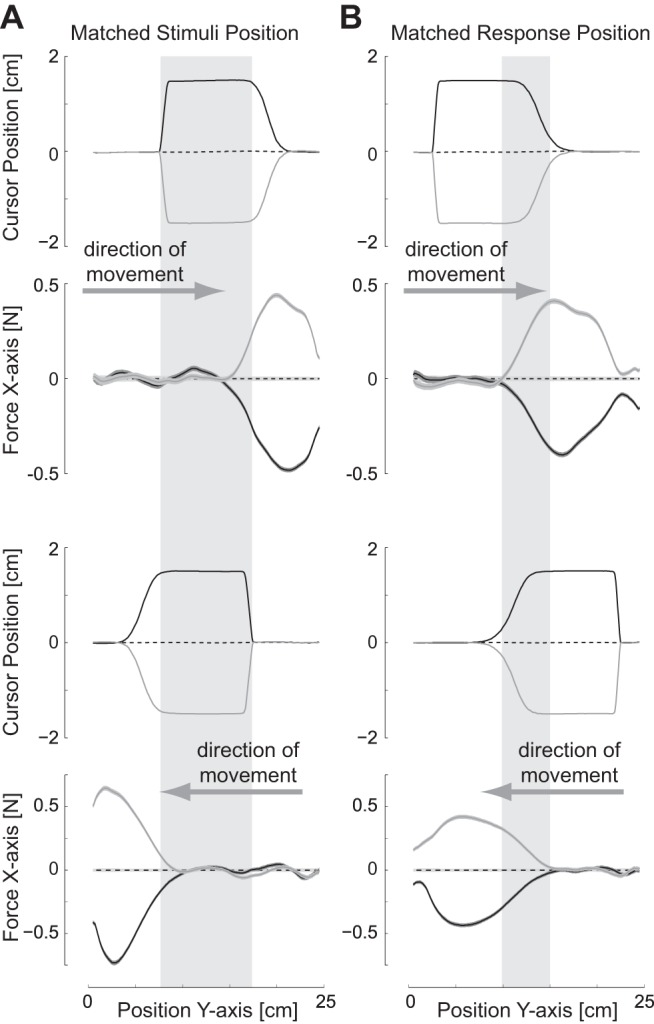
Comparison of the timing of perturbations in *experiment 1*. For each movement direction, the cursor position (*x*-axis) and the corresponding change in force against the channel wall (*x*-axis) are plotted as a function of the position in the *y*-axis. Mean and SE (shaded region) are plotted across all 24 subjects from *experiment 1*. *A*: forces during the 30% onset cursor perturbations (matched stimuli position) for the outward reaching (*top*) and inward reaching (*bottom*) movements. Shaded gray region demonstrates the matched positions (limb postures) over which the cursor perturbations in the outward and inward movements occur. *B*: forces during the 10% onset cursor perturbations (matched response position) for the outward reaching (*top*) and inward reaching (*bottom*) movements. Shaded gray region demonstrates the matched positions (limb postures) over which the force responses to the visuomotor perturbation in the outward and inward movements occur.

Subjects performed reaching movements while holding the handle of a robotic manipulandum (Fig. 1*A*). On random probe trials during normal reaching movements subjects were presented with a visual perturbation of the hand cursor while the hand itself was mechanically constrained to move within a channel to the target. These perturbations occurred either to the left or right of the hand trajectory at either 10% of the movement distance or 30% of the movement distance (Fig. 1*D*) in order to produce perturbations occurring at the same physical location on the outward and inward movements. The early perturbations produced a feedback response as measured by the force exerted against the channel wall occurring at the same physical location for both the outward and inward movements (Fig. 2*B*). In this condition, the physical location of the arm was similar in both directions of movements at the time of the feedback response. On the other hand, in case the onset of the perturbation was the relevant state to determine the feedback gain, the later perturbation led to the perturbation occurring at the same location for the outward and inward movements (Fig. 2*A*). By using both perturbations, the modulation of the involuntary visuomotor responses could be examined for matching physical locations of the arm both for the visual stimuli and for the reflex response to determine whether the feedback responses could be independently tuned for different reaching velocities (negative or positive) for the same physical location.

**Fig. 3.**
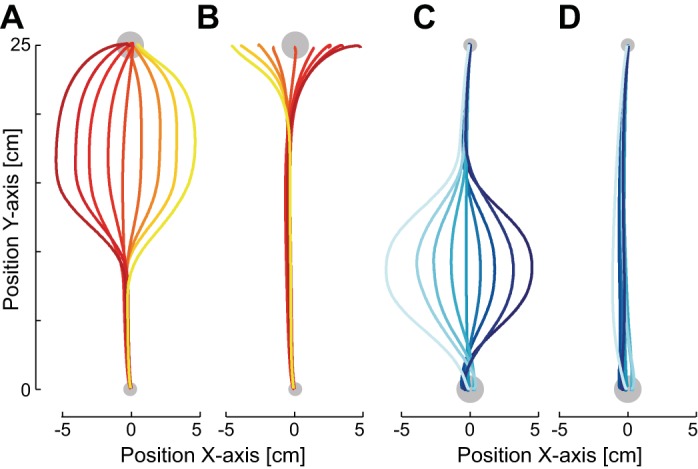
Kinematics in *experiment 1* for *group 1*. *A*: mean cursor trajectories for the 9 different trajectories for a single subject in the task-relevant condition during outward reaching movements. *B*: mean hand trajectories for the same movements are shown for the same subject, with the colors indicating the corresponding cursor motions. *C*: mean cursor trajectories for the 9 different trajectories for the same subject in the task-irrelevant condition during inward reaching movements. *D*: mean hand trajectories for the same movements.

In the initial half of the experiment (1,122 movements), both directions of movement were performed in the normal environment where the actual position of the hand was accurately represented by the visual feedback in all nonprobe trials. In the second half of the experiment, the outward reaching movements were performed in a task-relevant sensory discrepancy environment while the inward reaching movements were performed in a task-irrelevant sensory discrepancy environment (*group 1*; Fig. 1*C*) or vice versa (*group 2*). The hand kinematics and visual feedback for these movements are shown in Fig. 3. In the task-relevant environment subjects made corrective responses late in the movement to reach the final target (Fig. 3*B*), whereas in the task-irrelevant environment subjects did not respond to the visual motion, instead performing straight movements to the target (Fig. 3*D*).

**Fig. 4.**
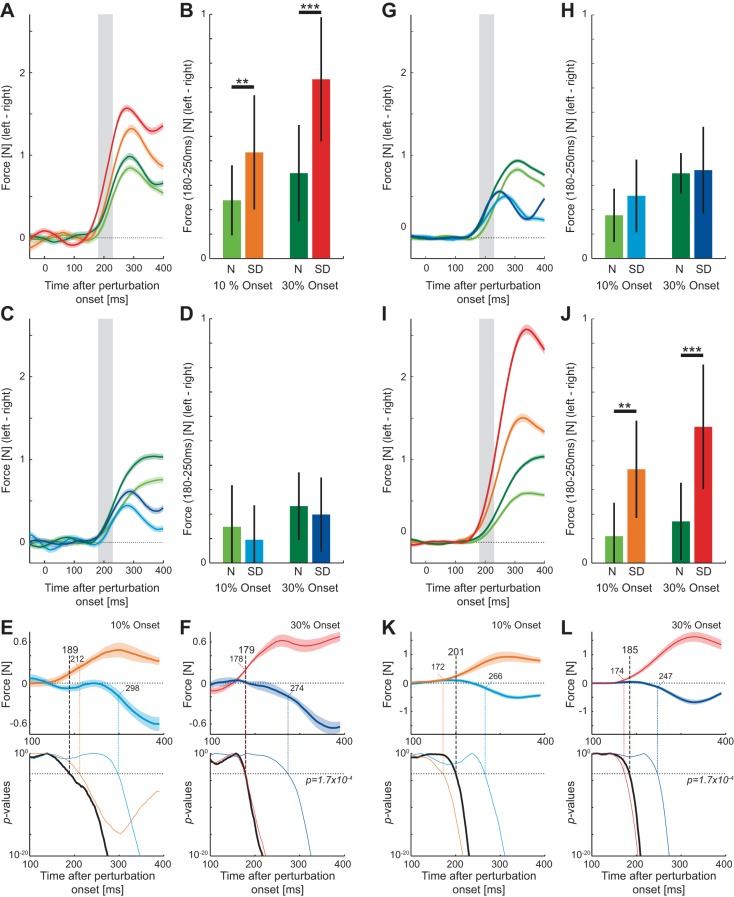
Visuomotor responses on probe trials in *experiment 1*. *A–F*: force responses for *group 1*, in which the outward movement was task relevant and the inward movement was task irrelevant. *A*: mean force response to visual perturbations (probe trials) in the normal and task-relevant visual environments across all subjects in the outward reaching movement. Responses in the first 5 blocks in each condition were not included. The response in the normal environment for both the 10% onset (light green) and 30% onset (dark green) perturbations and in the task-relevant visual environment for both the 10% onset (orange) and 30% onset (red) perturbations are shown as a function of time from the onset of the visual perturbation. Colored shaded regions indicate the SE across subjects. Gray bar illustrates the involuntary window (180–230 ms). *B*: mean ± SD force response over the involuntary interval for the outward reaching movements. Statistically significant differences between the conditions were tested with Tukey's honestly significant difference (HSD) post hoc test (**P < 0.005, ***P < 0.001). N, normal; SD, sensory discrepancy. *C*: mean force response to 10% onset (light color) and 30% onset (dark color) visual perturbations in the normal (green) and task-irrelevant (blue) visual environments during inward movements. *D*: mean ± SD force response over the involuntary interval for the inward movements. *E*, *top*: difference in force responses for 10% onset perturbations between the task-dependent environment and the normal environment for both the task-relevant (orange) and task-irrelevant (light blue) movement directions (zero line represents the force response in the normal environment, so that differences can be examined relative to this response). The difference between these 2 measures was examined every 1 ms with a *t*-test (Bonferroni corrected for multiple comparisons) to determine the time point where statistical differences between the 2 task-dependent environments first occurred. *Bottom*: *P* values for this comparison (black) as well as for each response relative to the normal environment (task relevant: orange; task irrelevant: blue) plotted as a function of the time after the onset of the perturbation. Dotted lines show the significance level and the time point at which significance was achieved for each of the comparisons. *F*, *top*: difference in force responses for 30% onset perturbations between the task-dependent environment and the normal environment for both the task-relevant (red) and task-irrelevant (dark blue) movement directions. *Bottom*: *P* values from the *t*-test comparisons. *G–L*: force responses for *group 2*, in which the outward movement was task irrelevant and the inward movement was task relevant.

The force responses to the visual perturbations (probe trials) were compared between the two stages of the experiments for the same reaching direction. For the first group of subjects, the force responses were relatively low in the normal environment but increased rapidly to a high level in the task-relevant sensory discrepancy environment for the outward reaching movements (Fig. 4*A*). The visuomotor responses were compared in the involuntary response interval (180–230 ms) to examine whether differences could be distinguished prior to the possible involvement of voluntary feedback changes (Fig. 4*B*). For both the 10% (matched response) and 30% (matched stimuli) onset perturbations, the forces were higher in the sensory discrepancy environment. These differences were examined with an ANOVA with main effect of environment type and subject as a random effect. The responses were found to be significantly larger in the task-relevant condition matched for both 10% onset perturbation (*F*_1,11_ = 13.805; *P* = 0.003) and 30% onset perturbation (*F*_1,11_ = 103.311; *P* < 0.001) locations.

Similarly, the responses were examined for the inward reaching movements (Fig. 4, *C* and *D*). While the initial force responses were similar in both the normal and sensory discrepancy environments, the response decreased in the task-irrelevant environment ∼300 ms after the onset of the visual perturbation (Fig. 4*C*). Again the responses were compared during the involuntary response time (180–230 ms) (Fig. 4*D*). By ANOVA, the responses were found to be not significantly different between the two conditions for both 10% onset perturbation (*F*_1,11_ = 1.151; *P* = 0.306) and 30% onset perturbation (*F*_1,11_ = 0.47; *P* = 0.507) locations.

The results from the above analysis suggest that the feedback forces were modulated independently for the outward and inward directions within the involuntary time window. To examine the time point at which such independent modulation occurs in the feedback responses, the difference in the force responses between the final sensory discrepancy environment and the initial normal environment was calculated every 1 ms throughout the movement for each subject (Fig. 4, *E* and *F*). *t*-Tests (Bonferroni corrected for multiple comparisons) were then used to test whether the difference in these responses between the outward movements (task relevant) and inward movements (task irrelevant) was significant at each time point. Significant differences in the responses were found starting from 189 ms (*t*_834_ = 3.803; *P* = 1.53e^−04^) for the 10% onset perturbations and from 179 ms (*t*_838_ = 3.971; *P* = 7.76e^−05^) for the 30% onset perturbations, well before the earliest voluntary change in feedback force (230 ms) for such visual perturbations (Franklin and Wolpert 2008). It is important to note that the onset times for the excitation of the response in the task-relevant environment (relative to the normal environment) is much earlier (10% onset: 212 ms; 30% onset: 178 ms) than the inhibition of the response found in the task-irrelevant environment (10% onset: 298 ms; 30% onset: 274 ms).

A second group of subjects performed the reverse experiment where the task-irrelevant sensory discrepancy occurred on the outward movement and the task-relevant discrepancy on the inward movement in the second half of the experiment. The results showed the same effects across all conditions as for *group 1*. The responses were found not to be significantly different in the task-irrelevant condition matched for either 10% onset (*F*_1,11_ = 3.059; *P* = 0.108) or 30% onset (*F*_1,11_ = 0.081; *P* = 0.782) perturbations (Fig. 4, *G* and *H*). However, the responses were found to be significantly larger in the task-relevant condition for both the 10% onset (*F*_1,11_ = 15.835; *P* = 0.002) and 30% onset (*F*_1,11_ = 34.56; *P* < 0.001) perturbations (Fig. 4, *I* and *J*). Again, *t*-tests (Bonferroni corrected for multiple comparisons) were used to determine whether there were significant differences in these responses between the outward movements (task irrelevant) and inward movements (task relevant). Significant differences in the responses were found starting from 201 ms (*t*_838_ = 3.84; *P* = 1.32e^−4^) for the 10% onset perturbations (Fig. 4*K*) and from 185 ms (*t*_838_ = 3.94; *P* = 8.89e^−5^) for the 30% onset perturbations (Fig. 4*L*), again well before the earliest voluntary response (230 ms) for such visual perturbations (Franklin and Wolpert 2008). Note again that these early differences are brought about by the excitation in the task-relevant (compared to the normal) condition rather than the inhibition in the task-irrelevant (compared to the normal) condition. The onset times in the task-relevant conditions were 172 ms (10% onset) and 174 ms (30% onset), whereas in the task-irrelevant they were 266 ms and 247 ms, respectively.

#### Experiment 2—effect of perturbation direction.

**Fig. 5.**
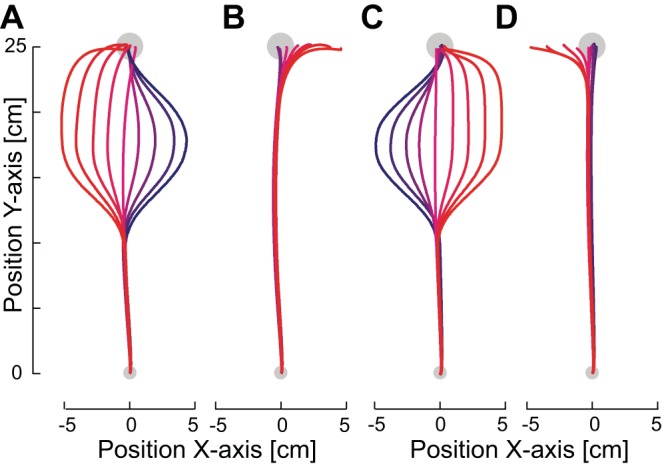
Kinematics in *experiment 2*. *A*: mean cursor trajectories for the 9 different trajectories for a single subject in the left-side task-relevant condition. *B*: mean hand trajectories for the same movements, with the colors corresponding to the cursor motion. *C*: mean cursor trajectories for the 9 different trajectories in the right-side task-irrelevant condition. *D*: mean hand trajectories for the same movements.

**Fig. 6.**
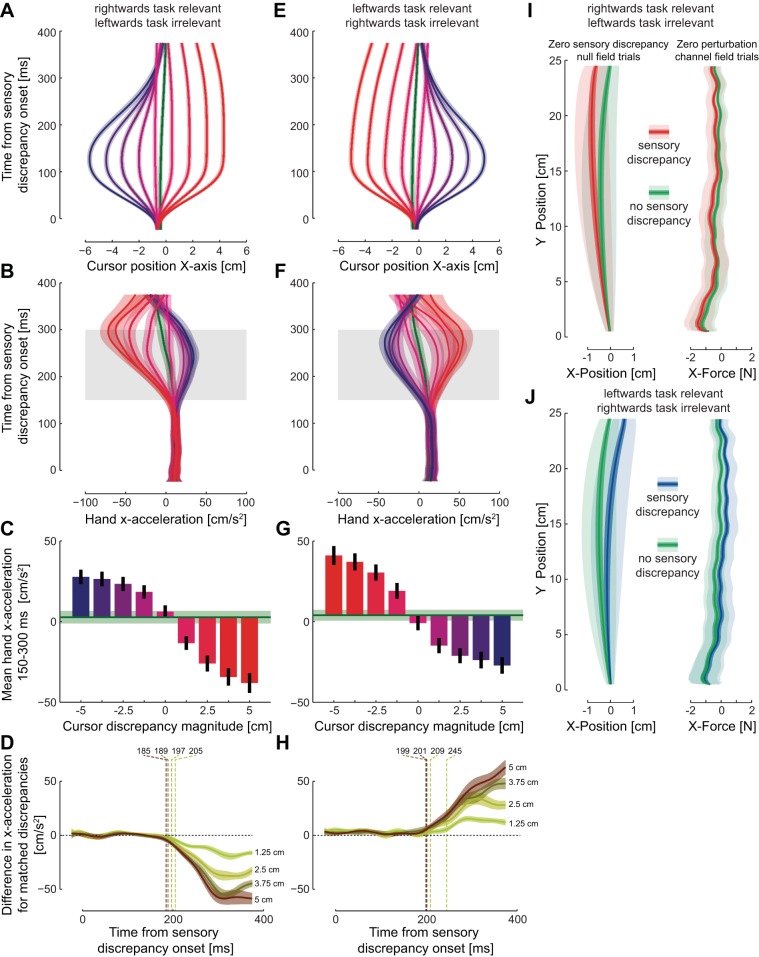
Corrective responses on the sensory discrepancy trials. *A*: mean (solid lines) and SE across subjects (shaded region) for the cursor position on the sensory discrepancy trials for the rightward task-relevant and leftward task-irrelevant environments. Green trace shows the unperturbed mean trajectory for the previous no sensory discrepancy environment. Values are plotted as a function of time from the onset of the sensory discrepancies. *B*: mean hand acceleration in the lateral direction for the conditions shown in *A*. *C*: mean hand acceleration over the interval 150–300 ms from the onset of the discrepancies. Green line and shaded region show the mean values for the initial no sensory discrepancy trials. *D*: difference in the hand *x*-acceleration between the sensory discrepancies of matched size for the conditions in *C*. The acceleration for the sensory discrepancy to the right (e.g., +5) was contrasted with the acceleration to the left for the same magnitude discrepancy (e.g., −5) to determine the onset time of any difference in the magnitude of these responses (dashed line). If the magnitudes were of equal size the difference would remain at zero. Vertical dashed lines indicate the time at which the difference was determined to be significantly different for each condition. *E*: cursor position for the leftward task-relevant and rightward task-irrelevant environments. *F*: lateral hand acceleration for the condition shown in *A*. *G*: mean hand acceleration over the interval 150–300 ms. *H*: difference in the hand *x*-acceleration between the sensory discrepancies of matched size for the conditions in *G*. *I*, *left*: comparison of the mean trajectories for the normal reaching trials in which the visual cursor matches the hand location (zero sensory discrepancy) in the no sensory discrepancy condition (green) and the rightward task-relevant and leftward task-irrelevant condition (red). Mean, SE, and SD are plotted as a function of the distance to the target. *Right*: comparison of the mean force exerted on the channel wall in the zero perturbation probe trials in the 2 conditions. *J*, *left*: mean trajectories for the normal reaching trials (zero sensory discrepancy) in the no sensory discrepancy condition (green) and the rightward task-relevant and leftward task-irrelevant condition (red). *Right*: mean force exerted on the channel wall in the zero perturbation probe trials.

In the second experiment, subjects made outward reaching movements to a target. During the first half of the experiment subjects performed these movements in the normal environment where the visual feedback matched the physical position of the hand (Fig. 1*E*), while during the second half of the experiment a sensory discrepancy environment was introduced (Fig. 1*F*). This sensory discrepancy environment was task relevant on one side of the reaching movement and task irrelevant on the other side (subjects experienced both variants of the sensory discrepancy environment on separate days, counterbalanced across subjects). The hand kinematics and visual feedback for these movements are shown in Fig. 6. Similar to *experiment 1*, when subjects had movements in which the visual path was task relevant they made corrective responses late in the movement to reach the final target, whereas when the visual path was task irrelevant subjects did not respond to the visual motion, instead performing straight movements to the target (Fig. 5, *B* and *D*).

The movements on these sensory discrepancy trials were examined to see any evidence for a change in the feedback responses (Fig. 6). The lateral hand acceleration changed ∼150 ms after the onset of the sensory discrepancies, with larger and more scaled responses to the task-relevant discrepancies than to the task-irrelevant discrepancies (Fig. 6, *B* and *F*). Such responses suggest that the subjects were modulating their feedback gains separately to either side of the reaching movement. This was examined in more detail by determining the difference in the size of the response for sensory discrepancies of matched magnitude but opposite sign (e.g., +5 vs. −5) relative to the undisturbed trajectory (Fig. 6, *D* and *H*). If the magnitudes of the acceleration were identical then the difference would remain at zero; however, for both cases the response in the direction of the task-relevant environment was significantly higher than for the task-irrelevant environment. The onset of the differences was examined with *t*-tests of the acceleration responses at each point in time, with significance determined at *P* < 0.05 (Bonferroni corrected for multiple comparisons). Seven of the eight comparisons showed onsets of the modulation of the responses occurring prior to the earliest voluntary response in force (230 ms; Franklin and Wolpert 2008). However, another modulation between the environments could also be seen. Specifically, the trajectory in the zero sensory discrepancy trials shifted between the initial no sensory discrepancy environment and the final sensory discrepancy environment. This was examined in detail over the entire movement, using both the trajectory on the identical zero sensory discrepancy trials and the lateral force on the zero perturbation channel trials (Fig. 6, *G* and *H*). Subjects shifted their mean trajectory by 6.2 mm (at the end of the movement) toward the task-irrelevant side of the environment. This adaptation of the mean trajectory has two consequences, the first to assist the correction of the task-relevant shift in the cursor position and the second to compensate (in advance) for the incompletely suppressed responses to the task-irrelevant shifts in the cursor position. Thus a shift in the mean trajectory assists with the compensation to this environment. This trajectory shift was also apparent on the force exerted on the channel trials with mean differences of 0.26 N between the two conditions. Together, these results demonstrate that subjects adapted to the environment, at least partially, through trajectory modulation and also suggest that they modulated their feedback responses. However, these sensory discrepancy trials provide no estimate of the baseline feedback gain in the normal trials and also introduce difficulty in determining the detectable onset time of the cursor shift because of their smooth onset. Therefore we use an independent measure of feedback responses on probe trials to assess any changes in the feedback gains.

**Fig. 7.**
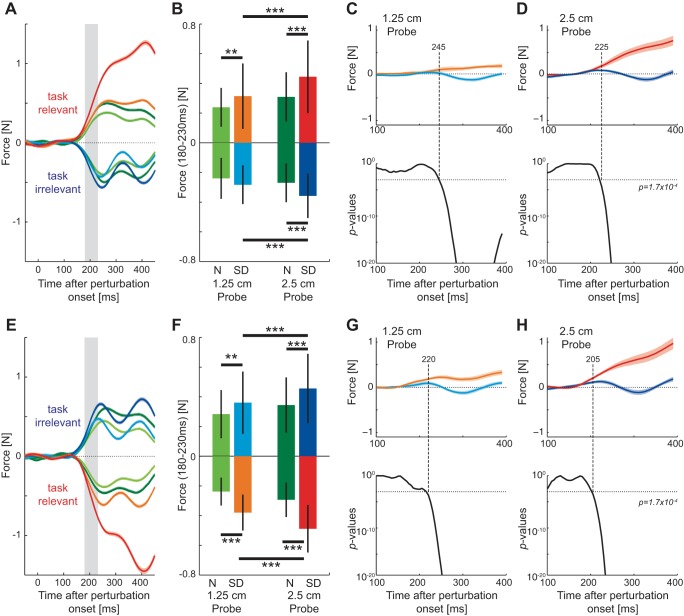
Visuomotor responses on probe trials in *experiment 2* in both the normal and sensory discrepancy environments. *A*: mean force response to the small (1.25 cm) and large (2.5 cm) visual perturbations in the normal and right-side task-relevant environments across all subjects. The first 5 blocks in each condition were not included. The colors correspond to the environment (normal: green; task relevant: red; and task irrelevant: blue) for both the 1.25-cm (light colors) and 2.5-cm (dark colors) probe trials as a function of time after perturbation onset. *B*: mean ± SD force response over the involuntary interval (180–230 ms). Statistically significant differences between the conditions were tested with Tukey's HSD post hoc test (**P < 0.005; ***P < 0.001). *C*, *top*: difference in force responses for 1.25-cm perturbations between the sensory discrepancy environment and the normal environment for both the task-relevant (orange) and task-irrelevant (light blue) perturbation directions. The difference between these 2 measures was examined every 1 ms with a *t*-test (Bonferroni corrected for multiple comparisons) to determine the time point where differences between the 2 perturbation directions first occurred. *Bottom*: *P* values for these comparisons plotted as a function of the time after the onset of the perturbation. Dotted line shows the level at which significance was considered. *D*: difference in force responses for 2.5-cm perturbations between the sensory discrepancy environment and the normal environment for both the task-relevant (red) and task-irrelevant (dark blue) perturbation directions. *E*: mean force responses for the left-side task-relevant environment experiment. *F*: mean ± SD force responses across subjects over the involuntary interval. *G*: change in force responses between the task-relevant and task-irrelevant perturbations relative to the original normal environment for the 1.25-cm probe trials. *H*: change in force responses between the task-relevant and task-irrelevant perturbations relative to the original normal environment for the 2.5-cm probe trials.

On random trials, in order to estimate the feedback gains, a visual perturbation of the cursor occurred for 250 ms in which the location of the cursor was shifted to either the left or right by 1.25 or 2.5 cm (probe trials) (Fig. 1*G*). These perturbations were used to examine whether subjects could independently regulate the gain of the feedback responses to the left and right of the reaching movement on the same movement. The force responses to the visual perturbations (probe trials) were compared between the two stages of the experiments for each variant of the sensory discrepancy environment separately. The force responses to the visual perturbation during the normal environment were similar in size to the right and left of the movements (Fig. 7, *A* and *E*), with the response to the 2.5-cm perturbation slightly larger than that to the 1.25-cm perturbation. Once the sensory discrepancy environment was introduced, perturbations into the task-relevant side of the environment produced larger force responses for both the 1.25-cm perturbation and 2.5-cm perturbation (Fig. 7, *A* and *E*). On the other hand, perturbations into the task-irrelevant side of the environment produced little change in the force response for either the 1.25-cm or the 2.5-cm perturbation.

The visuomotor responses were compared in the involuntary response interval (180–230 ms) to examine whether differences could be distinguished prior to the possible involvement of voluntary feedback changes (Fig. 7, *B* and *F*). The magnitudes of the force responses were compared separately for perturbations to the right and left. On the experimental day in which the task-relevant sensory discrepancy occurred on the left hand side of the straight reaching movement (Fig. 7, *A* and *B*), an ANOVA with main effects of perturbation size (2 levels: 1.25 cm and 2.5 cm) and environment (2 levels: normal and sensory discrepancy) and random effect of subject was performed for the perturbation to the left (positive force responses in Fig. 7*A*). Significant differences were found for the size of the perturbation (*F*_1,15_ = 93.714; *P* < 0.001) and the environmental condition (*F*_1,15_ = 15.161; *P* = 0.001) and an interaction between these two (*F*_1,15_ = 14.374; *P* = 0.002), demonstrating that the task-relevant environment increases the gain of the visuomotor responses. Finally, for direct comparisons across the conditions an ANOVA was run with a main effect of condition (4 levels) and random effects of subjects and planned comparisons between conditions either with the same perturbation size and/or same environment. After a significant main effect (*F*_3,45_ = 24.1; *P* < 0.001), differences were examined with Tukey's HSD post hoc test (shown in Fig. 7*B*). The same analysis was performed for the perturbations to the right (negative force responses in Fig. 7*A*). The ANOVA found significant main effects for both perturbation size (*F*_1,15_ = 29.989; *P* < 0.001) and environment (*F*_1,15_ = 12.330; *P* = 0.003) as well as a significant interaction effect between these two (*F*_1,15_ = 7.554; *P* = 0.015). Again, after a significant main effect across all perturbations (*F*_3,45_ = 16.157; *P* < 0.001), individual differences were examined with post hoc tests (Tukey's HSD) as indicated in Fig. 7*B*.

Identical analysis was performed on the responses on the other day of experiments, where the task-relevant environment was on the right-hand side of the reaching movement (Fig. 7, *E* and *F*). For perturbations to the left (task-irrelevant side), an ANOVA with main effects of perturbation size and environmental condition found significant effects for perturbation size (*F*_1,15_ = 39.334; *P* < 0.001) and environmental condition (*F*_1,15_ = 8.252; *P* = 0.012) but no significant interaction between these effects (*F*_1,15_ = 1.877; *P* = 0.191). The appropriate individual differences were investigated with a different ANOVA, with significant post hoc results plotted (Fig. 7*F*) after the significant main effect (*F*_3,45_ = 4.208; *P* < 0.001). For perturbations to the right (task-relevant side), an ANOVA with main effects of perturbation size and environmental condition found significant effects of perturbation size (*F*_1,15_ = 39.954; *P* < 0.001) and environmental condition (*F*_1,15_ = 120.489; *P* < 0.001) and a significant interaction between these effects (*F*_1,15_ = 13.175; *P* = 0.0.002). Again, after a significant main effect (*F*_3,45_ = 73.999; *P* < 0.001), the appropriate individual differences were investigated with post hoc tests and plotted (Fig. 7*F*).

The results from the above analysis suggest that the feedback responses were modulated independently for perturbations to the left and right of the movement direction within the involuntary time window. However, significant increases were also found for many of the perturbations into the task-irrelevant side of the environment. To examine whether there was independent modulation of the feedback responses on each side of the movement and determine at what time such independent modulation occurs, the difference in the force responses between the normal environment and the sensory discrepancy environment was calculated every 1 ms within a movement for each subject (Fig. 7, *C*, *D*, *G*, and *H*). *t*-Tests (Bonferroni corrected for multiple comparisons) were used to test whether the difference in these responses was significant between the responses to the left (task relevant) and the right (task irrelevant) at each time point. Significant differences in the responses were found starting from 245 ms (*t*_1438_ = 3.3894; *P* = 7.19e^−4^) for the 1.25-cm perturbations (Fig. 7*C*) and from 225 ms (*t*_1438_ = 4.09; *P* = 4.57e^−5^) for the 2.5-cm perturbations (Fig. 7*D*). Similar times were found for the opposite sensory discrepancy environment, where the responses were significantly larger to the right (task relevant) than to the left (task irrelevant) (Fig. 7, *G* and *H*). Significant differences in the responses were found starting from 220 ms (*t*_1438_ = 3.74; *P* = 1.89e^−4^) for the 1.25-cm perturbations and from 205 ms (*t*_1438_ = 3.63; *P* = 2.97e^−4^) for the 2.5-cm perturbations (Fig. 7, *G* and *H*). These differences (across all subjects) occurred just prior to the earliest detectable voluntary change in feedback force found for a single subject (230 ms) (Franklin and Wolpert 2008) for such visual perturbations for three of the four cases. As such, we suggest that these changes in modulation are occurring within the involuntary responses rather than through a voluntary correction, although the low-pass filtering effects of the muscles limit the discriminatory power of these responses. To examine this in more detail, the responses during this involuntary period were examined in the muscle activity.

**Fig. 8.**
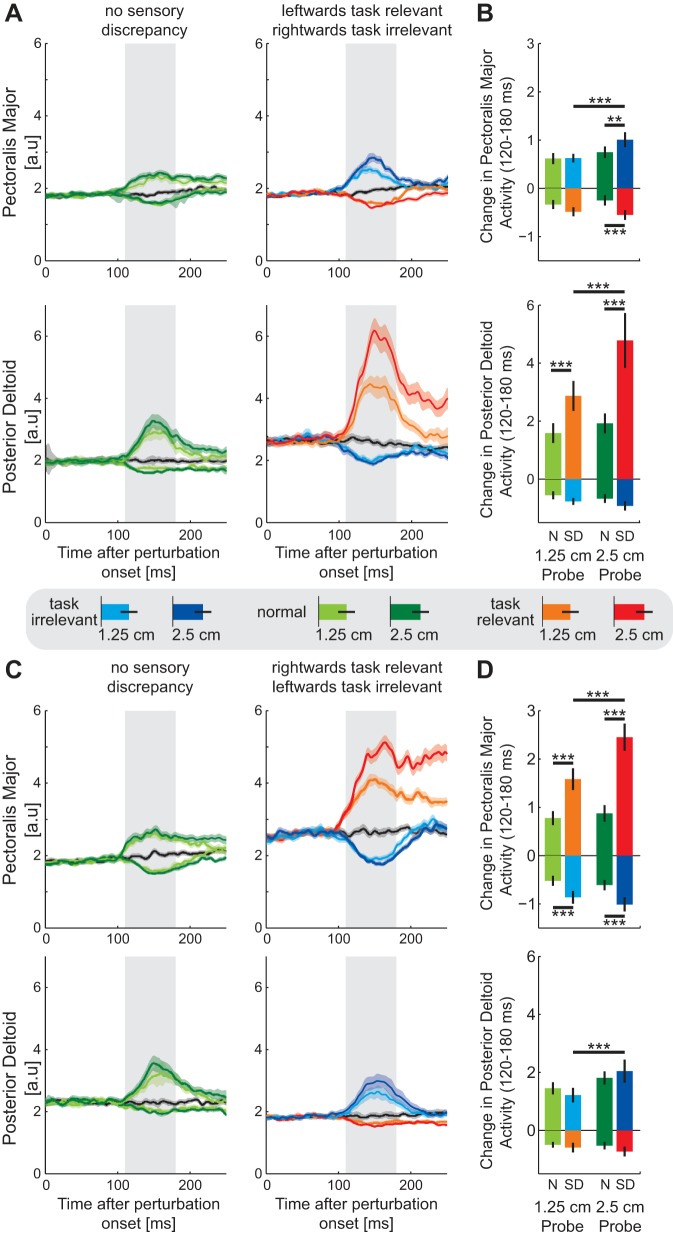
Muscular responses to visual perturbations (probe trials) during *experiment* 2. *A*: mean (solid line) ± SE (shaded region) muscle activity [arbitrary units (a.u.)] in the pectoralis major (*top*) and posterior deltoid (*bottom*) for perturbations in the normal (*left*) and leftward task-relevant sensory discrepancy (*right*) environments. Responses to the perturbations into the task-relevant part of the environment are shown in orange (1.25 cm) and red (2.5 cm), into the task-irrelevant part of the environment in light (1.25 cm) and dark (2.5 cm) blue, and into the normal environment in light (1.25 cm) and dark (2.5 cm) green. The response to the zero visual perturbation condition is shown in black. Shaded gray bar indicates the involuntary response interval (120–180 ms). *B*: mean ± SD muscular response over the involuntary interval (180–230 ms) for the leftward task-relevant sensory discrepancy environment. Statistically significant differences between the conditions were tested with Tukey's HSD post hoc test (**P < 0.005; ***P < 0.001). *C*: mean (solid line) ± SE (shaded region) muscle activity in the pectoralis major (*top*) and posterior deltoid (*bottom*) for perturbations in the normal (*left*) and rightward task-relevant sensory discrepancy (*right*) environments. *D*: mean ± SD muscular response over the involuntary interval for the rightward task-relevant sensory discrepancy environment. ***P < 0.001.

The muscular responses to the visual perturbations of the cursor position were examined in the posterior deltoid and pectoralis major (Fig. 8) and quantified over the involuntary time period (120–180 ms). Examining the left-side task-relevant environment first (Fig. 8, *A* and *B*), an ANOVA was performed separately for the perturbations to the right and left for each muscle. Examining perturbations into the task-relevant side of the environment, there was a significant main effect in the pectoralis major (*F*_3,45_ = 8.15; *P* < 0.001), with larger inhibition for the task-relevant compared with normal environment in the large perturbation (*P* < 0.001). In the posterior deltoid, after a significant main effect (*F*_3,45_ = 14.392; *P* < 0.001), post hoc analyses demonstrated larger responses in the task-relevant environment than for the same perturbation in the normal environment for both perturbation sizes (both *P* < 0.001). Moreover, in the task-relevant environment, the 2.5-cm perturbation produced a much larger response than the 1.25-cm perturbation (*P* < 0.001), whereas there was no significant difference between these two sizes in the normal environment (*P* = 0.452). Perturbations presented into the task-irrelevant side of the environment also produced significant changes relative to the normal environment. In the pectoralis major, after a significant main effect by ANOVA (*F*_3,45_ = 7.551; *P* < 0.001), post hoc tests indicated a general increase in response for the large perturbation in the task-irrelevant environment compared with either the large perturbation in the normal environment (*P* = 0.007) or the smaller perturbation in the task-irrelevant environment (*P* < 0.001). In the posterior deltoid, although there was a significant main effect (*F*_3,45_ = 4.103; *P* = 0.012), post hoc tests indicated no significant differences in the relevant comparisons.

Similar responses can be found in the other environment (right-side task-relevant environment; Fig. 8, *C* and *D*). Perturbations presented into the task-relevant side showed significant differences in the pectoralis major by ANOVA (*F*_3,45_ = 35.047; *P* < 0.001). Post hoc analyses demonstrated larger responses in the task-relevant environment than for the same perturbation in the normal environment for both perturbation sizes (both *P* < 0.001). Moreover, in the task-relevant environment, the 2.5-cm perturbation produced a much larger response than the 1.25-cm perturbation (*P* < 0.001), whereas there was no significant difference between these two sizes in the normal environment (*P* = 0.869). In the posterior deltoid no significant changes were found for any of the perturbations (*F*_3,45_ = 1.826; *P* = 0.156). However, perturbations presented into the task-irrelevant side of the environment also produced significant changes relative to the normal environment. In the pectoralis major, after a significant main effect by ANOVA (*F*_3,45_ = 8.105; *P* < 0.001), post hoc tests indicated a general increase in inhibition in the task-irrelevant environment compared with the normal environment (both *P* ≤ 0.01) but no changes between the small and large perturbation sizes in either environment (both *P* > 0.35). In the posterior deltoid, after a significant main effect (*F*_3,45_ = 6.53; *P* = 0.001), post hoc tests indicated an increase in the response to the two perturbation sizes in the task-irrelevant environment (*P* < 0.001), with no other significant differences in the appropriate comparisons. Overall, although significant effects are seen in these early involuntary intervals for both the task-relevant and task-irrelevant environments, the difference between these two, with increased excitation and consistent and appropriate changes in feedback gain in the task-relevant environment, can be clearly seen in the responses.

## DISCUSSION

The results of this study provide clear evidence that the brain can learn to modulate the gain of the visuomotor response (motor output to perturbations of the visual location of the hand during movement) for a single limb posture depending on the direction of either the movement or the perturbation. Specifically, we have exploited the finding that this visuomotor response magnitude is affected by the statistical properties of the visual environment (Franklin and Wolpert 2008) to determine whether the visuomotor responses could be independently modulated either to the direction of hand motion or to the direction of the perturbation. The first experiment demonstrated that subjects could appropriately learn to produce larger feedback responses in outward reaching movements while alternating with smaller feedback responses in the inward movements (or vice versa) when these two movement directions were paired with task-relevant and task-irrelevant perturbations, respectively. The second experiment demonstrated that the feedback responses could be independently tuned to the left and right of the movement, with gain increasing on only one side in which task-relevant perturbations occurred. We suggest that these results indicate that subjects are able to learn to fractionate their feedback responses as a function of the state of the limb and the state of the perturbation.

The first experiment showed differential feedback modulation for alternating reaches in two directions. Interestingly, the early feedback responses in the task-irrelevant condition were not significantly different from those in the normal environment, in contrast to our previous study (Franklin and Wolpert 2008). One explanation is that the normal environment may be considered task irrelevant, as the random probe trials contain visual perturbations that themselves are a type of sensory discrepancy. However, this is true for both the present study and the 2008 study, so it is unlikely to contribute to the difference between them. It has previously been shown that learning to inhibit the visuomotor feedback gain is much slower than increasing the gain (Franklin and Wolpert 2008) and produces a smaller difference. Thus any competition (or interference) between excitation and inhabitation of the feedback pathways in the alternating movements would produce a larger influence on the responses in the task-irrelevant condition. This experiment also produced responses to the task-relevant condition that appeared to lead the normal responses by a fixed delay rather than a simple gain increase under some (but not all) conditions (Fig. 4). Although there are variations in the response onset time (Fig. 4), these were not consistent across conditions, suggesting that it is unlikely to result from actual differences in the latencies. Moreover, any significant early difference was maintained throughout the response time, indicating that this difference was maintained. Any differences in these responses (either in timing or magnitude) are unlikely to be due to an effect of either attention or a startlelike response. This is because attention has been shown not to modulate the responses to cursor perturbations (Reichenbach et al. 2014). Similarly, startlelike responses, if induced, would first contribute to changes in the force in 105 ms (Valls-Solé et al. 1995) rather than 140 ms and, more critically, should be induced across all conditions (as the perturbation trials are identical) rather than being expressed only in a task-dependent manner based on the surrounding nonprobe trials.

The visuomotor feedback responses that in this study range from 0.5 to 1 N have been shown to vary in magnitude up to 1.5 N (Dimitriou et al. 2013; Franklin et al. 2012; Franklin and Wolpert 2008; Reichenbach et al. 2013, 2014) depending on the particular environment in which subjects are moving and the task they are performing. Although these magnitudes may appear small, it is worth considering that they contribute a change in EMG activity and end-point force similar to those arising from stretch reflexes (Franklin et al. 2008). Moreover, even the addition of visuomotor responses, stretch reflex responses, and limb stiffness will produce between 3 and 5 N of force in response to perturbations of 8 mm during whole-arm reaching movements (Burdet et al. 2000). This can also be estimated from the measured end-point stiffness of the arm during null-field reaching movements, which ranges in the lateral direction from 200 to 400 N/m (Franklin et al. 2003, 2004, 2007). A perturbation of 1 cm would therefore be expected to result in a change in force of 2–4 N total from all contributions including muscle stiffness and feedback responses. Thus even this relatively small change in end-point force produces a significant contribution of the total response during a reaching movement, and its effect on the movement of the limb can be seen both in the acceleration changes on the sensory discrepancy trials (Fig. 6) and in other studies (Brenner and Smeets 2003; Wijdenes et al. 2011, 2013).

There are extensive studies demonstrating the modulation of feedback responses to the properties of the task that subjects perform (Franklin and Wolpert 2011; Pruszynski and Scott 2012). For example, studies have demonstrated the modulation of both stretch reflex and visuomotor responses to changes in the environmental dynamics (Akazawa et al. 1983; Cluff and Scott 2013; Doemges and Rack 1992a, 1992b; Franklin et al. 2012; Kimura and Gomi 2009), the visual statistics (Franklin and Wolpert 2008), the shape of the target (Knill et al. 2011; Nashed et al. 2012), and when the two limbs manipulate a single object (Diedrichsen 2007; Dimitriou et al. 2012; Omrani et al. 2013). Similarly, studies have shown that the long-latency stretch responses are modulated appropriately to the limb dynamics (Kurtzer et al. 2008; Lacquaniti and Soechting 1986; Pruszynski et al. 2011a; Soechting and Lacquaniti 1988), producing required compensatory responses even when the muscle is not perturbed. However, most of these studies have examined the feedback responses for a particular task, where during the time period that the subject performs the task the feedback gains are either globally increased or decreased appropriately.

Very few studies have examined the changes in feedback responses within a single task, i.e., simultaneously increasing and decreasing the feedback gains appropriately within a single movement or task. Similar to experiments in locomotion where the gains vary with the phases of the step cycle (Capaday and Stein 1986; Sinkjaer et al. 1996), it has been shown that the visuomotor gains vary as a function of the distance to the target (Dimitriou et al. 2013; Liu and Todorov 2007; Wijdenes et al. 2011). All of these responses vary according to the task requirements (stage of locomotion or distance to target), meaning that they also vary with the limb posture. It is possible, therefore, to consider most of these state-dependent results as task-dependent expression of the spinal circuitry (Pearson 2000; Schieppati 1987). In contrast, here we have shown not only that these responses are prestored functional responses varying only with the limb posture but that they can be learned appropriately for the particular environment, tuned independently (upregulated and downregulated) for identical limb postures with different velocity or perturbation direction states. This demonstrates that visuomotor feedback gains can also be learned independently to either side of the movement, similar to what has been previously demonstrated for stretch feedback responses when learning to compensate for novel dynamics (Kimura and Gomi 2009) or to avoid objects during reaching (Nashed et al. 2014). This work also extends the findings of Knill et al. (2011), showing that these feedback gains can be modulated differentially to either side of the movement and not only between perturbations in direction and extent.

Previous studies of visuomotor feedback responses have shown clear changes in the feedback magnitudes from the earliest detectable responses (Franklin et al. 2012; Franklin and Wolpert 2008). For example, when the sensory discrepancy was either task relevant or task irrelevant, the differences were apparent from the initial response (140 ms onward), leading to clear differences over the involuntary window used in this study (Franklin and Wolpert 2008). However, in this study, although the differences between the task-relevant and task-irrelevant conditions were clear at later intervals, the earliest responses were not statistically different between these two conditions. It is only closer to 180 ms (*experiment 1*) or 200 ms (*experiment 2*) that these differences become statically significant. This suggests that fractionating the response based on movement or perturbation direction may require extra processing time. Such a possibility is similar in some sense to the rapid motor responses to muscle stretch: different functional responses at different delay times (Crago et al. 1976; Pruszynski et al. 2011b). For stretch responses, although the initial short-latency response is not modifiable [except on a long timescale (Wolpaw et al. 1983)] and only exhibits gain scaling (Marsden et al. 1976; Pruszynski et al. 2009), the longer-latency responses can be modulated according to the task demands (Dimitriou et al. 2012; Kurtzer et al. 2008; Nashed et al. 2012, 2014; Shemmell et al. 2009) and may involve cortical circuits similar to those involved in voluntary motor control (Pruszynski et al. 2011a; Zuur et al. 2009, 2010) as well as subcortical components (Grey et al. 2001; Lewis et al. 2004).

However, unlike the stretch reflex, the pathways involved in producing the rapid visuomotor responses are unknown. Some evidence suggests the involvement of the superior colliculus, which is strongly involved in visually guided motion (Courjon et al. 2004; Gahtan et al. 2005; Song et al. 2011; Sprague and Meikle 1965), whereas other evidence has suggested the premotor cortex, specifically PMv (Jackson and Husain 1996; Kurata 1994) or parietal cortex (Diedrichsen et al. 2005). One possibility is that the later differentiation of the feedback gains involves a neural circuit different from the earliest response, either allowing for further processing of the information or integrating further sensory feedback and task goals into the response. However, it is important to note that the responses during this interval (up to 230 ms) still cannot be eliminated despite explicit task commands (Day and Lyon 2000; Franklin and Wolpert 2008).

The subjects in *experiment 2* not only modified their feedback responses but also shifted their mean trajectory toward the task-irrelevant side of the sensory discrepancy environment. This change in the movement execution acted to both partially compensate for the possible maintained task-relevant perturbations (therefore requiring less corrective response) and partially compensate (in advance of the perturbation) for the elicited feedback response in the task-irrelevant perturbations. The only condition in which this change in trajectory was not appropriate was the zero sensory discrepancy trial. Thus the subjects found a shift that helped to compensate but still allowed this trial to end in the target location. We believe this shift in the trajectory is another example of the sensorimotor control system reoptimizing the trajectory to ensure task completion (Izawa et al. 2008; Nagengast et al. 2009).

One of the key results of this work is the demonstration that these rapid feedback responses can be adapted simultaneously to multiple environmental conditions during a single movement. This demonstrates several important features of the feedback gains. The first feature is that these feedback gains are continually learned and adapted to the tasks being performed. Indeed, several studies have shown the gradual learning of feedback gains as subjects learn environmental dynamics (Cluff and Scott 2013; Franklin et al. 2012). The second feature is that these responses can be fractionated appropriately rather than simply upregulated or downregulated. Although on a single movement only one feedback gain was actually expressed (because of our applied perturbation), the feedback gains for the left and right visuomotor responses had to be appropriately set, as the direction of the perturbation was not known in advance (*experiment 2*). The sensorimotor control system can, therefore, prepare an appropriate pattern of feedback gains for the environment and tune these feedback responses to the environment as part of the adaptation process for the performance of skilled movement.

## GRANTS

This work was financially supported by the Wellcome Trust (WT091547MA and WT097803MA), the Biotechnology and Biological Sciences Research Council (BBSRC; BB/J009458/1) and the Royal Society Noreen Murray Professorship in Neurobiology (to D. M. Wolpert).

## DISCLOSURES

No conflicts of interest, financial or otherwise, are declared by the author(s).

## AUTHOR CONTRIBUTIONS

Author contributions: D.W.F. and D.M.W. conception and design of research; D.W.F. and S.F. performed experiments; D.W.F. analyzed data; D.W.F. interpreted results of experiments; D.W.F. prepared figures; D.W.F. drafted manuscript; D.W.F., S.F., and D.M.W. edited and revised manuscript; D.W.F., S.F., and D.M.W. approved final version of manuscript.
